# Observations on Diffused Cellular Inflammation, with Cases

**Published:** 1827-01

**Authors:** Henry Earle

**Affiliations:** St. Bartholomew's Hospital.


### THE LONDON
### Medical and Physical Journal.

N° 335, VOL LVII.] JANUARY, 1827. [N° 7, *New Series.*

For many fortunate discoveries in medicine, and for the detection of numerous errors, the world is indebted to the rapid circulation of Monthly Journals; and there never existed any work, to which the Faculty, in Europe and America, were under deeper obligations, than to the Medical and Physical Journal of London, now forming a long, but an invaluable, series.—RUSH.

### ORIGINAL PAPERS,
AND
CASES OBTAINED FROM PUBLIC INSTITUTIONS AND OTHER AUTHENTIC SOURCES.

### CELLULAR INFLAMMATION.

*Observations on diffused Cellular Inflammation, with Cases.* Being the Substance of a Clinical Lecture delivered by HENRY EARLE, F.R.S. &c. (ST. BARTHOLOMEW'S HOSPITAL.)

UNDER the title of diffused cellular inflammation, I wish to consider those cases which have been classed under the general names of phlegmonous and traumatic Erysipelas. Names are of but little importance, provided the ideas we attach to them be distinct, and not likely to be confounded. It is on this account that the accurate definition of a disease is essential, for an incorrect one will give rise to wrong ideas, which will infallibly lead to erroneous practice. I object to the term phlegmonous erysipelas, because I think that it is likely to be confused with common acute erysipelas, and because I consider erysipelas essentially as an affection of the skin; whereas the disease under consideration exerts its influence principally on the subcutaneous tissue and fascia. It is true that this is accompanied with an erysipelatous redness of the skin, but this is rather to be considered as an effect of the changes which are taking place in the subcutaneous cellular tissue, than as the original primary affection. This distinction may not at first sight appear very important, but in a practical point of view it will be found of the greatest consequence.

During the last few months, several cases of this affection have occurred at St. Bartholomew's Hospital, in most of which the practice recommended by Mr. Copland Hutchison has been successfully employed. There are at the present time two cases in a convalescent state, which bear testimony in favour of this method of treatment. Before proceeding to relate these cases, it will be well to consider the peculiar characteristic symptoms which mark this affection, and offer some observations on the nature of the treatment, and the objects proposed to be attained by it.

I have stated that the seat of this disease is in the subcutaneous cellular tissue: it consists in a rapidly diffused acute inflammation in this tissue, to which no bounds are set, and which, if not arrested quickly, terminates in most extensive suppuration and sloughing of the cellular substance and fascia, which relieves itself, if the patient survives, by large and uncontrollable sloughing of the integuments.

I have known, in the short space of thirty-six hours, the whole integument of an upper or a lower extremity involved in one extensive sloughing abscess. This affection most frequently takes place in the extremities: in Mr. C. Hutchison's cases, it almost constantly occurred in the lower extremities, and was often produced by the irritation caused by the friction of coarse trowsers, wetted with sea water, on old indolent ulcers. I have more frequently met with it in the upper extremity. It has generally followed punctured wounds or severe bruises upon the elbow, and lacerations of the integuments of the hands or fingers. In several instances I have known it occur after punctured wounds of joints, or in the neighbourhood of joints, which have been attempted to be closed. In the majority of cases, it is accompanied in the early stages with well-defined inflammation of the absorbents; but these soon become so involved in the general tumefaction of the limb, that they cannot be traced. This swelling takes place with great rapidity and to great extent, accompanied with a dusky red appearance of the surface. In the earlier stages, there is a peculiar elastic feel in the integuments, which afford much greater resistance than in common œdema. If the integuments be divided at this period of the affection, the fat and cellular substance cuts with great crispness, and the wound gapes much. After a time, the sensation imparted to the touch is somewhat analogous to the crepitation of emphysema: it has a peculiar boggy feel; and now we find, on cutting down, that the work of destruction has taken place.

The constitution participates much with the local affection. The secretions are either all suspended or deranged. The circulation is much hurried; the pulse hard, contracted, and frequent, and after a time irregular. The nervous system is greatly disturbed; the countenance becomes most anxious, and features contracted. There is great and constant vigilance; or, if the patient drops asleep, he awakes in alarm, or in a state of delirium. When the cellular membrane has extensively sloughed, the greatest prostration of strength accompanies it, and a state resembling the worst form of irritative sympathetic fever often closes the scene.

The treatment best suited to this formidable affection, (which, if adopted in time, seldom fails to arrest its progress,) is the one which has been recommended by Mr. Copland Hutchison, in a paper published in the Medico-Chirurgical Transactions; to the real merits of which I can bear most ample and satisfactory testimony. This treatment consists in making very free longitudinal incisions, if possible before suppuration has taken place, through the swollen inflamed integuments down to the fascia or muscles. The vessels of the skin should be allowed to bleed freely, and even encouraged by warm fomentations; the limb should then be enveloped in a warm bread-and-water poultice. A large dose of calomel, antimony, and opium, should be administered; and after some hours the patient should be freely purged with senna and salts, or some other active aperient. If the incisions be made sufficiently large and deep, the relief is very speedy, and it is seldom requisite to repeat them. In a few hours I have witnessed the subsidence of tension and pain, the nervous system tranquillised, and the secretions restored.

Objections have been raised against this practice, on the ground of the impropriety of inflicting fresh injury on parts already inflamed. This certainly appears very specious; but facts are obstinate, and many, which are well authenticated, can be produced to prove the fallacy of the arguments and the efficacy of the practice. Again, it has been contended that this mode of treatment was rude, and not adapted to civil life. To me this argument appears so puerile as hardly to merit a reply. If by bold and decided measures we can arrest the progress of a most formidable and rapidly destructive disease, surely it is our duty to employ them for the benefit of all alike:—disease makes no such distinctions of rank, and why should the surgeon?

If any additional reason were required in support of this practice, besides the rapid and almost certain relief which it affords, we shall, I think, at once be reconciled to it, when we consider that by these very incisions we are most probably preserving the integuments from sloughing, and rendering it unnecessary to make far more extended wounds, which we should otherwise be compelled to have recourse to, after allowing the disease to run its course. And further, in making these incisions at an early period of the complaint, we are enabled to select those parts for dividing the integuments where there is least hazard of wounding any vessels or nerves of importance. The incisions should be made from three to six inches in length, in the long axis of the limb, which will best remove the distressing tension.

The rationale of this treatment appears to be the relief afforded to the immediate seat of the disease, which consists in a state of acute inflammation of the cellular membrane, a part endued with low powers of vitality.*

I am well aware of the heavy responsibility which a surgeon incurs in adopting, in the early stage, this bold and apparently severe treatment, before the necessity for it may be obvious to the patient or his friends; but this is a responsibility which must often attach to medical men in the performance of their duty, and it is one which no man competent to the exercise of his profession should ever shrink from. Let the surgeon first qualify himself for the performance of his painfully anxious vocation, by patient and laborious investigation of original facts, and then let him discharge his duty fearlessly, and to the best of his abilities. I should be very sorry to be misunderstood, or to mislead others. Let it not be supposed that I am an advocate for making incisions in every case of erysipelas attended with swelling of the limb. Certainly not. But, in this particular form of cellular inflammation, I feel perfectly satisfied that the practice recommended is beneficial, and is indeed the only method of treatment which is capable of arresting the progress of the disease.

This conviction rests principally on the evidence of cases which have occurred at St. Bartholomew's Hospital, several of which are of very recent date. Before proceeding to detail some of these facts, I will add one more remark. As the treatment recommended in these cases is bold and severe, it is of the utmost importance to discriminate accurately between this peculiar affection and the more common forms of erysipelas. To obtain this knowledge, we must make observations at the bedside of the patient: no verbal description, however correct, can convey that impression which "the

* We often have opportunities of witnessing the comparative vitality of the skin and subcutaneous tissue, not only in this disease, but in herpetic ulcers and other complaints, where this structure perishes much more extensively than the superincumbent skin.

faithful eye" and touch will alone impart. The disease, once fully recognised, cannot easily be mistaken.

I subjoin some cases which have lately occurred, and which have been drawn up by the gentlemen who conducted the treatment under my superintendence.

CASE I.—*Cellular Inflammation of Hand and Arm, from the Bite of a Dog.*

John Wilson, ætatis thirty-four, of a strong constitution and a free liver, was admitted into Harley's Ward, on the 18th of June, 1826, with an inflamed hand from the bite of a dog. As there was no reason to believe the dog was mad, it was treated as common phlegmonous inflammation.

The pulse being full and strong, ninety-six, with great pain in the hand and increased heat, sixteen ounces of blood were taken from the arm, and twenty leeches applied to the back of the hand. He was ordered to take a dose of calomel and jalap immediately, and saline medicines every four hours; and to apply a bread-and-water poultice to the hand.

June 19th.—The hand, though much improved, is still greatly inflamed, but not much pain in it. Twenty leeches were applied to the hand; to continue with the medicine as before.

From this time till July 2d, an interval of thirteen days, the hand gradually improved. He then, without being able to account for the change, complained of a pain in his head and sickness: he had a good deal of fever; the hand and forearm were swollen and inflamed. He was ordered an emetic; eighteen leeches were applied to the hand and arm; he was bled to sixteen ounces, and was ordered a saline mixture with antimony, to take every four hours.

July 3d.—Hand better and pain diminished, but the inflammation of the forearm continues, and has run up the absorbents nearly to the axilla. On examining the back of the hand attentively, a slight fluctuation was felt. Pulse 100, full and strong. An incision was made, about two inches and a half in length, on the back of the hand, which gave vent to a quantity of matter that had been secreted beneath the fascia. He was bled to twelve ounces, and twenty leeches were applied to the arm.

At nine P.M. he had no pain in the hand, but complained more of the arm. Pulse ninety-six, full and strong. Ten ounces of blood were taken away, and twenty leeches applied to the arm.

4th.—Both hand and arm much improved.

5th.—The forearm is more inflamed and more swollen to-day. He was ordered some calomel and jalap, and fourteen leeches to be applied to the forearm. Absorbents inflamed to the axilla.

6th.—The forearm more swollen than it was yesterday; pulse 100, full and strong; tongue furred. An incision was made, about five inches in length, from the olecranon towards the hand, not with the expectation of finding matter, but to give relief to the extreme tension which was present. The wound bled about half a pint. Twenty leeches were applied to the front of the arm.

The following day, the arm was very much improved in every respect, and continued so till the 10th, when he had an attack of fever, caused by using too much exertion in endeavouring to get out of bed, which gave rise to a fresh attack of inflammation in the arm. His pulse being full and strong, ninety-eight, with a hot dry skin, he was bled to twenty ounces; and half a scruple of Potass Nitras added to each dose of his mixture. After this he daily improved; and, on the 18th, he was ordered the Unguent. Zinci, to be applied to the hand and arm. He was now put on meat diet, and remained in the hospital till August the 1st, when he was discharged cured.

In this case there was an unusual degree of inflammatory action, the pulse remaining strong and full after very active depletion. The incision afforded great relief, but, in consequence of the wound not bleeding freely, it was necessary to apply more leeches. The incision, I have no doubt, prevented extensive suppuration. The wound healed very readily.

Case II. *Cellular Inflammation of the Leg.*

Joseph Potter, ætatis twenty-six, of a strong constitution, and rather a free liver, was admitted, August 10th, into Colston's Ward, with an ulcer in his leg. A poultice was applied, and for a few days it seemed inclined to heal. On the 28th, he had an attack of fever, accompanied by all the usual symptoms in an excessive degree. The ulcer was much inflamed and very painful. He was bled to twenty ounces, and ordered saline medicines to take every three hours. The following morning, the fever had much abated, but the ulcer and inflammation of the leg were the same.

August 29th.—The fever had returned with increased violence; the leg was much swollen, and the inflammation increased. Sixteen ounces of blood were taken from the arm. To continue with the medicines as before.

31st.—The fever had somewhat abated, but the inflammation had greatly increased, extending up the absorbents: the leg was much swollen, extreme tension was present, and the pain most violent. An incision, from four to five inches in length, was made on the posterior part, through the fat and cellular tissue, down to the fascia. A small artery was divided; and the wound bled to about half a pint. The relief afforded was so great, that five minutes afterwards he complained of little or no pain, except from the smarting of the incision. He was ordered a cathartic, a bread poultice to the leg, and an evaporating lotion to the thigh. At nine P.M. he was free from pain, and the leg looked better.

September 1st.—Had slept well the fore part of the night, but towards morning became very restless, and complained of a slight pain in the head. The leg was greatly improved, the wound looked well, and the inflammation had left the absorbents. He was ordered some Ol. Ricini. At seven P.M. the pain in the head had increased; pulse full and frequent, skin hot. Twenty ounces of blood were drawn from the arm; to continue with the medicines as before.

Sept. 2d.—Had passed a good night; was in no pain, the leg much improved, and the wound discharged a healthy pus. From this time it improved daily, as also did his general health.

On the 6th, his foot and ankle were swollen and inflamed. Twelve leeches were applied, and a bread poultice afterwards. It was better on the following day. On the 9th, twelve more leeches were applied, and in two or three days it was quite well.

On the 15th, he was put on a better diet; the leg was bandaged; and on the 8th instant he was discharged cured.

This case speaks so strongly for itself, that it hardly requires a comment. It is to be remarked that, in both the cases above related, the inflammation of the absorbents readily subsided after the incisions.

Case III. *Cellular Inflammation following Venesection.*

James Bannister, ætatis thirty-six, was admitted into Pitcairn's Ward, August 6th. He stated that, four days previously, he had been bled for a pleurisy. Two days after, pain and tumefaction of the arm took place, preceded by severe rigors. When admitted, the arm presented a very inflamed swollen appearance, the tumefaction extending nearly to the shoulder, with inflammation of the absorbents to the axilla, and an erysipelatous blush spreading over the chest. He was suffering under a high state of irritative fever, with headache and a much loaded tongue. Calomel and tartar emetic were ordered for him, with saline aperients, and tepid poppy fomentations to the arm.

On the following day, the tension of the arm having increased, and the constitutional symptoms not at all abated, very free incisions were made on the forearm and arm. Suppuration and sloughing of the cellular membrane had taken place near the wounded vein. The wound in the upper arm bled freely. In the evening, he was much relieved. All inflammatory symptoms rapidly subsided. Extensive sloughs of the fascia were drawn away from the forearm; after which the wounds granulated, and healed readily under the use of Sulphate of Quinine, and occasional doses of Extr. Colocynth. Comp. and Calomel.

In this case the incisions were not made sufficiently early to prevent the destruction of the fascia and cellular membrane in front of the forearm, but the immediate stop which they put to the progress of this affection in the upper arm was well marked.

Case IV. *Cellular Inflammation of the Hand and Arm, following a Wound of the Forefinger.*

James Hailes, ætatis twenty, a chair and sofa maker, was admitted into Colston's Ward, Saturday, October 21. He had struck his forefinger against a man's tooth on the previous Saturday. He had been a free liver, and was rather intoxicated at the time of the accident. The wound rapidly inflamed, and was very painful. The hand had been fomented and poulticed, and he had taken saline aperients previously to his admission. Diffused cellular inflammation took place up the arm, and the hand became greatly swollen. When admitted, there was a large sloughy abscess at the back of the hand, which was freely opened by the gentleman who attended him; who also made a small puncture on the arm, which bled freely. The integuments, to some extent, were gangrenous at the back of the hand.

I saw the patient a few hours after his admission, and found him with a most anxious countenance and hard contracted pulse, with dark fur on his tongue, and great prostration of strength. The absorbents of the upper arm were inflamed to the axilla; the integuments of the forearm were of a dusky red colour, and imparted a boggy feel when pressed. The tension appeared in some degree to have subsided, which is the case when suppuration has taken place. I immediately made a very free incision through the integuments along the edge of the ulna, to the extent of five inches. The cellular membrane was sloughy, and some pus escaped. The wound bled very freely, and he lost about thirty ounces of blood in a short time. He was rather faint, but felt much relieved in the space of a few hours. Calomel, antimony, and opium, were given to him at night.

The following morning, all appearance of inflammation of the absorbents had subsided; the skin of the forearm was cool, and all pain had ceased. From this time he experienced no bad symptoms, and the patient is now convalescent.

I come now to speak of a case which is peculiarly interesting to surgeons, and more especially to those who are engaged in anatomical pursuits. It is the only instance in which I have employed this treatment to diffuse cellular inflammation arising in consequence of a morbid poison. It occurred to one of my dressers, who in part drew up the following particulars.

Case V. *Cellular Inflammation of the Hand and Arm, following a Wound received in Dissection.*

Mr. C. E. B—, ætatis twenty-two, of sanguine temperament and a free liver, on the 21st of April, in opening the cranium of a man who died of gangrenous erysipelas after injury to the head, pricked the middle finger of his right hand with a small spicula of bone: the wound was so small that it did not bleed. On going to bed, after entertaining some friends at dinner, and taking his share of the wine, his hand became afflicted with pain, which rapidly increased, with an intense burning sensation in the finger. He applied cold water, but, finding no relief, he had recourse to a bread-and-water poultice; notwithstanding which, he could obtain no sleep. On the following morning, eight leeches were applied, and a deep incision was made near the seat of the injury, but without any benefit: the inflammation continued to spread over the hand.

I saw him about the middle of the day, at which time the first phalanx of the finger had perished. Distinct lines of inflamed absorbents could be traced up the arm, especially at its outer surface, but no tenderness or pain was felt any where except in the finger and hand, and there it was of the most intense and burning nature. Leeches were again applied, and the poultice repeated; his bowels were freely moved with calomel and tartar emetic, and senna and salts mixture. The pain in the hand continued unabated, and could only be tolerated by applying the coldest water.

On the morning of the 23d, after a sleepless night, the hand and arm had a puffy œdematous appearance, although the redness of the absorbents was diminished. The tongue was furred, and pulse hard and frequent; skin hot and parched, and countenance much distressed. Sixteen ounces of blood were taken from the arm, and cold constantly applied to the hand. Opii gr. ij. Pulv. Antim. gr. iv. were given at night.

24th.—He had passed a restless night, with constant state of vigilance. The arm was more swelled, and extremely painful; pulse 120, but softer. Opium was given in three-grain doses during the paroxysms of pain.

25th.—The night had again passed without sleep, and at intervals there was delirium. The arm was more puffy, but the pain was rather diminished. Oleum Ricini was given to obviate costiveness, and the opium continued. The following night passed tranquilly, but without sleep.

On the 26th, the arm was again very painful, and much swollen. It was enveloped in a bread poultice, made with a strong watery solution of opium. His bowels were open; his pulse frequent, but not strong; tongue covered with much brown fur; and he was more disposed to delirium, with a most anxious sunken countenance. Thirty drops of Guttæ Nigræ were given at night, in addition to the former pills of opium.

On the 27th, there was no very sensible change. Still no sleep at night.

On the 28th, he was visibly much worse. The inflammation had extended to the deltoid muscle on the outer side, but did not appear to have reached the axilla on the inner side; nor was there any pain at this part, or disposition to extend over the pectoral muscle. From the insertion of the deltoid downwards, the whole arm was as tense as possible, and felt remarkably firm when pressed. The colour was a dusky red, rather more vivid at the upper margin. He had been delirious all night, and appeared rather comatose. His countenance was shrunken, wild, and ghastly. He was so weak that he could not sit up in bed; his pulse was irregular, feeble, and fluttering; his tongue covered with thick brown fur. It was evident that he could not long survive under these circumstances, and, although I had no evidence in favour of the practice in a similar case, I resolved to make large and deep incisions, provided it met with Mr. Lawrence's concurrence, who was so obliging as to visit him with me. Mr. L. entertained the same view of the case, and in his presence I made three deep incisions,—one commencing a little above the insertion of the deltoid, and more to the outer side, which extended down to the olecranon; a second, about six inches long, from just below the olecranon to the wrist; and a third, about three inches in extent, on the inner side of the forearm. No suppuration or sloughing was apparent, but the wounds gaped much, and the fat was very firm and granular. The wound at the outer side of the forearm bled very freely, to the extent of from thirty to forty ounces; after which the limb was enveloped in a large bread-and-water poultice. In the evening, when I saw him, his countenance was much improved; and his pulse was steady, soft, and full, about eighty beats in the minute. The pain had nearly subsided, except in his finger. He was ordered a dose of calomel and jalap, and to continue the opium after its operation.

29th.—He had passed the night tranquilly, but without sleep. His countenance was much improved, and comparatively cheerful; pulse stronger, and quite soft; arm quite easy; skin flaccid and pale.

30th.—He was nearly the same as yesterday; still no tranquil sleep could be obtained. In the evening there was great restlessness, with slight wandering. Two grains of opium and six of camphor were given to him at night, which, for the first time since the receipt of the injury, procured repose. He slept tranquilly for four hours, and awoke perfectly collected, and with the arm quite easy. When I paid my visit, I found him greatly improved in every respect. Suppuration had taken place at the wounds. His bowels were moved with Infus. Sennæ and Tinct. Jalapæ; and, after the operation of the medicine, he took solid food with much relish. The opium and camphor were repeated at night.

From this time he continued to go on most favourably: very copious healthy suppuration came readily away from beneath the whole integuments of the upper and fore arm; but no sloughing took place of the cellular membrane or fascia. By lighter dressing and bandaging, the whole rapidly filled up and skinned over. In a few days he was ordered the Sulphate of Quina in Infus. Rosæ, and was allowed to take a mutton-chop and some claret.

Nothing particular occurred during the subsequent treatment, except the formation of abscesses at the back and front part of the hand, which required to be freely opened: during the formation of these, the constitution again suffered some excitement. The extremity of the wounded finger was also very painful, until the lateral ligaments were divided, and the dead portion removed. The finger remained swollen for a considerable time, and was the last part to heal. He subsequently went out of town, when he rapidly regained his health and strength.

I consider this case as particularly valuable, as it is the first instance, as far as I am aware, of the application of the practice to this form of disease. The rapidly destructive consequences which occasionally follow the most trifling wounds received in dissection, and particularly in the examination of recent bodies, are well known to the profession. Many most interesting and melancholy details of the premature death, after severe suffering, of some of the brightest ornaments of our profession, have been recorded in medical works, and especially in the able Treatise of Mr. Travers on Constitutional Irritation. The present case differs in many respects from most of those recorded, and more nearly resembles that form of diffused cellular inflammation which I have endeavoured to explain.

That nothing short of the severe measures which were adopted would have saved the life of this young gentleman, I believe every person who witnessed the case was perfectly convinced. Should a similar case ever again fall under my observation, I would certainly not hesitate so long before adopting this practice, as I firmly believe that the extensive suppuration might have been prevented, and the sufferings of the patient greatly curtailed, by its more prompt employment.

Since the lecture on the above subject was delivered, another striking example has occurred, the subject of which is at present in St. Bartholomew's Hospital.

Case VI. *Cellular Inflammation following Fracture of the Tibia.*

Richard Titcomb, ætatis thirty-five, was admitted November 10th, with comminuted fracture of the lower part of the tibia, and superficial laceration of the integuments in front of the leg above the fracture. The injury had been inflicted by the falling of a puncheon of rum. He was intoxicated at the time of his admission, and it appeared that he was much in the habit of drinking raw spirits. The fracture was reduced, and the limb placed on the side.

The case went on tolerably well for some days. On the 13th, the fractured bone appeared displaced by the action of the muscles, and I advised its being placed on the heel in a fracture-box.

On removing the splints, the wound looked sloughy, and the limb was much swelled and inflamed.

On the 14th, the inflammation had extended to the knee. He had passed a restless night, and he had considerable fever. Calomel and Antimony, and Haust. Sennæ Comp. were ordered, and a bread-and-water poultice applied.

On the 15th and 16th, I did not see the patient, but find, from the daily report which was kept by Mr. WATSON, that no material change took place until the night of the 16th, when all his symptoms became much aggravated. When I visited him on the 17th, I found that he had been delirious in the night, and had again displaced the fracture. The whole limb was immensely swollen; the superficial wound of the skin was sloughy; there was obscure fluctuation on the inner side of the tibia above the fracture, and a boggy feel around it; the absorbents were inflamed up to the groin, and a dusky inflammation extended beyond the knee; his countenance was flushed; tongue parched; pulse ninety-six, and rather feeble. I made a free incision on the inner side of the tibia, and gave exit to some matter, and exposed the sloughy cellular membrane. A further incision was made, about four inches long, on the outer side of the leg, which was very tense and inflamed, but no suppuration had yet taken place at this part. The wounds bled about eight or ten ounces. He was ordered calomel and jalap to open the bowels, and opium at night.

On the 18th, I found that he had passed another bad night. The pain in the leg was diminished, and the appearance of the integuments around the second incision was much improved, being pale and comparatively loose. The integuments in front of the tibia had sloughed to some extent, and the sloughy fascia was very apparent through the first incision. As the tension and inflammation of the inner side of the leg had not been sufficiently relieved by this incision, another was made below it, to the extent of four inches; in making which, a superficial vein was divided, which bled very profusely. To maintain the limb in the best position, and prevent the necessity for any motion, he was placed on one of my double inclined beds, and the foot fixed to a footboard.

The following day, (the 19th,) I found him greatly improved: he had passed a good night; the inflammation of the absorbents had disappeared, and the limb was only labouring under the effects of the previous mischief. Copious suppuration and extensive sloughs were the consequence of this; but the latter have all separated, leaving a healthy granulating surface, and the former is gradually diminishing under mild astringent dressing and bandages.

It has since been necessary to make some minor incisions on the instep and above the ankle; but the case, on the whole, is making very satisfactory progress.

Dec. 11th.—Soon after the above report was drawn up, the patient's health suffered materially; the granulations over the front part of the leg became glossy and smooth; and it was evident that the wound communicated with the comminuted fracture, and that no attempt at union had taken place. The integuments over the ankle and foot were quite hollow, and discharged a thin sanies at the openings which had been made. From the situation of the fracture, it was very probable that the fracture extended into the ankle-joint. Under these circumstances amputation of the leg was deemed adviseable, from a fear that the patient's constitution would sink under the continued drain and irritation.

The operation was performed on Saturday, the 9th of December, and was more tedious than usual, in consequence of the very firm adhesions which had taken place between the cellular membrane and fascia, and from the longitudinal incisions having extended nearly as high as the knee, both at the inner and outer side of the leg. Being very desirous of preserving the knee-joint, I carefully dissected up the integuments, after making a circular incision, and, by removing the spine of the tibia, in the manner recommended by ASSALINI, there was sufficient skin to cover the bones and meet together in a horizontal line. The appearance is not so neat as the more perpendicular stump, but, as far as I can at present judge, the case is likely to terminate well.

In this case, I think, if earlier and freer incisions had been made, when the inflammation of the absorbents and tension first supervened, much mischief might have been prevented. —In addition, I beg to observe, that in no one instance have I ever had occasion to regret making early incisions, although in several, as in this last case, I have conceived that delay has been productive of more serious consequences, and have induced the necessity for more severe measures in the sequel.

Although it has been necessary to remove the limb in this case, in consequence of the extent of the injury and the state of the patient's constitution, I do not consider that this at all militates against the practice of free incisions in cellular inflammation: on the contrary, it is a strong additional fact in support of it, as the patient was fast sinking under the disease when the incisions were made, and, but for the practice which was adopted, would never have survived to undergo amputation; as, of course, such an operation was quite inadmissible in the state in which I have described him to have been. I believe that every person who witnessed this case was satisfied that the man's life was preserved by the free incisions, and that he was brought into a state favourable for operation by their timely employment.*

* See a note from Mr. Earle in our Intelligence: it was received too late to embody it in the paper.—ED.

I am happy in having it in my power to subjoin a case, with the particulars of which I have been favoured by Mr. Warry, house-surgeon to St. Bartholomew's. The bold treatment which was employed I have no doubt preserved the patient's life; and, had it been adopted at an earlier stage to less extent, it would probably have arrested the progress of the mischief, and greatly reduced the severity of the subsequent treatment. It is interesting however, in showing how rapidly amendment will follow these free incisions, and with what perfect impunity they may be employed. In no one instance have I known the cut edges ulcerate or slough, except where the subjacent cellular membrane had extensively perished, and the integuments were in a state approaching to gangrene at the time of making the incision.

VII. *Case of Cellular Inflammation, in which an Incision was made, which extended from an inch below the great Trochanter to within an inch and a half of the Ankle.* (Bridgewater Infirmary.)

September 14th, 1822.—Elizabeth Parsons, ætatis twenty-seven, a young woman of a plethoric habit, was suddenly seized with a violent pain on the outer side of her right knee, whilst engaged in her usual occupation, that of house-maid in a gentleman's family. This pain continued for two days to increase, during which time there came on considerable swelling and inflammation, extending up the thigh and down the calf of the leg, attended with much fever and other constitutional disturbance. She was confined to her bed, was cupped and had leeches applied, and suitable medicines given.

On the sixth day, it was discovered, on examination, that matter had formed on the outer side of the knee, where the patient first complained of pain. It was therefore judged expedient to open the abscess; which was accordingly done by making a small puncture with an abscess-lancet, by which about four ounces of thick pus were evacuated. This, however, did not afford her the anticipated relief. The inflammation of the leg and thigh went on. Poultices and fomentations were applied, and, although the discharge from the puncture was considerable, the pain and constitutional disturbance continued: the latter, indeed, rather increased.

On the tenth day, a probe, twelve inches in length, was passed into the wound in a direction downwards, and it was found to extend to within an inch and a half of the external malleolus. It was then taken out, and its course directed up the outer side of the thigh; and in this direction the extent of the sinus could not be reached. The surgeon, considering a free incision as the most probable mode of relieving the constitutional symptoms, as well as of checking the sloughing of the cellular membrane, proceeded to lay open the entire extent of the sinus downwards, to within an inch of the external malleolus. The whole of the cellular membrane of the leg was found to be in a sloughing state.

On the following day, a free division of the sinus, extending upwards, was made to within an inch of the great trochanter of the thigh. The immense extent of the wound gave to it a very formidable appearance, particularly as all the cellular membrane was in a sloughing state, so that the finger could, in some parts of the thigh, be nearly passed round between the muscles and integuments. The patient was supported with wine and a generous diet.

Before two days had elapsed after the operation, the benefit resulting from this free division of parts was manifest, both locally and constitutionally; every thing assumed a most favourable aspect; and within a day of ten weeks from the division of the sinuses, she left the infirmary with the wound perfectly healed, with the exception of a small place about the size of a sixpence, and an œdematous state of the limb.

19, *George-street, Hanover-square*; *December* 11*th*, 1826.

